# Application of Interval Analysis to Assess Concrete Cover Degradation in Accelerated Corrosion Tests

**DOI:** 10.3390/ma16175845

**Published:** 2023-08-26

**Authors:** Faustyn Recha, Kseniya Yurkova, Tomasz Krykowski

**Affiliations:** 1 Faculty of Architecture, Civil Engineering and Applied Arts, Academy of Silesia, Rolna 43, 40-555 Katowice, Poland; faustyn.recha@wst.pl; 2 Department of Mechanics and Bridges, Faculty of Civil Engineering, Silesian University of Technology, Akademicka 5, 44-100 Gliwice, Poland; kseniya.yurkova@polsl.pl

**Keywords:** corrosion, FEM, elastic–plastic, cover cracking, durability

## Abstract

This paper presents the application of interval algebra in affine formulation to assess damage propagation in test reinforced concrete elements subjected to accelerated corrosion of rebar, taking into account the uncertainty of parameters. Corrosion interactions were captured by introducing the interval tensor of the velocity of volumetric strain. Analysis of the model for limit values of velocities of volumetric strains ($\inf\left({\dot{\bar{\varepsilon }}}^{V}\right)$ and $\sup\left({\dot{\bar{\varepsilon }}}^{V}\right)$) using the finite element method for locally and gradient-formulated concrete models with degradation, elastic, and elastic–plastic was conducted using ANSYS and ATENA software. Computer calculations were performed assuming a parameter uncertainty of 0, 10, and 20%. The results of the calculations were compared with the results of detailed tests of elements subjected to accelerated corrosion of reinforcement using an electrolyzer with full monitoring of the electrical parameters of the system. The obtained results of the calculations were verified using the Monte Carlo method, treating the model parameters as random variables with a uniform distribution.

## 1. Introduction

The evaluation of the cracking time and degradation of reinforced concrete cover in accelerated corrosion tests has some specific features that differ from analogous issues related to natural reinforcement corrosion [1,2]. These differences are mainly related to very high values of the intensity and density of the corrosion current [1,3]. The nature and kinetics of the process affect the composition of corrosion products and make their composition dependent on the intensity of electrical phenomena [4]. In accelerated corrosion tests, the value of the electrochemical equivalent of iron is also different, and in the case of natural processes, it can be assumed to be analogous to that for pure iron $k_{{\mathrm{Fe}}^{2+}}$, given a charge number of the reaction equal to two.

From the point of view of mechanics, the process of damage evolution in the cover as a result of an accelerated corrosion process is similar to natural corrosion. As a result of the current flow, iron ions are carried into the solution, and corrosion products are formed in the first phase, tightly filling the pore spaces in the transition layer with increased porosity (ITZ interface transition zone) [5]. In the next phase, microcracks develop within adjacent pore spaces—the so-called corrosion product accumulation area (CAR) [6]—which influences the intensity of the impact of corrosion products on concrete cover. There are a number of different models describing the process of propagation of damage in the cover as a result of corrosion of reinforcement. A broad overview of analytical models can be found in [7]. For computer models, the effects of corrosion products are accounted for by introducing internal volumetric strains. This type of approach can be found, among others, in papers relating to the uniform distribution of products on the circumference of the rebar [8], non-uniform distribution [9], modeling by imposing displacement fields on contact elements [10], or approaches introducing a substitute tensor of volumetric strains [11,12] (in coupled description).

The difficulty of evaluating the propagation of damage caused by corrosion is the uncertainty of the parameters that characterize the course of the corrosion phenomena. Methods based on the probabilistic approach are not easy to implement due to the difficulty in determining the probability density function. In the group of papers on the probabilistic approach, one can distinguish items concerning the following: the impact of the probability density function on the failure probability of an element, as well as the reliability description of the safety of degrading elements under reinforcement corrosion over time [13]; in the broader context in the cycle [14,15,16], probabilistic assessment of damage propagation in the cover of reinforced concrete elements in relation to elements subjected to chloride corrosion [17]; microbial, climatic, and fatigue effects [18,19]; and the application of Bayesian methods to account for uncertainties in various nonlinear material models by correlating the results of FEM calculation with experimental results [20].

In the case of complex nonlinear calculations based on FEM in which uncertainties refer to various model parameters, such as material parameters, the only effective probabilistic computational method is a simulation approach such as the Monte Carlo method. A very in-depth analysis of this type of approach to a broad class of problems of nonlinear structural mechanics can be found in [21]. A serious disadvantage of this type of approach is the very high computational cost associated with solving multiple problems for various randomly selected parameters.

Alternative approaches to capture uncertainty include methods based on fuzzy numbers [22], interval arithmetic [23], and affine numbers, which are free from the problem of overestimation of the solution [24]. Such approaches also capture uncertainties that are not probabilistic. The application of interval methods in the mechanics of materials and structures can be found, among others, in papers concerning the use of a combined interval and probabilistic approach to the analysis of structural reliability [25]; the formulation of a method for solving the affine systems of equations, along with applications for FEM analysis of structures, Ref. [26]; or the formulation of a novel finite element method to limit conservatism affecting classical interval analysis in relation to linear problems of mechanics [27]. An application of interval methods in relation to the description of volumetric strains caused by the growth of corrosion products is presented in [28].

In this paper, the proposed approach [28] to the evaluation of damage propagations in elements subjected to accelerated corrosion tests, taking into account affine numbers, was modified and validated. The interval algorithm for determining increments of volumetric strains was adapted to the problems of evaluating accelerated corrosion tests and further extended. The interval approach was applied to the calculation of complex, nonlinear local and nonlocal models describing the degradation of concrete test specimens. For the purpose of verifying the calculations, experimental tests were carried out on reinforced concrete elements, which were subjected to accelerated corrosion of reinforcement [29]. The test results were used to verify the approach to estimating the width of crack opening, taking into account the uncertainty of the model parameters. The load was a time-varying increase in the interval tensor of volumetric strains caused by the impact of corrosion products on the concrete of the cover. The limits of corrosive increments of volumetric strains were calculated as follows: lower ($\inf\left({\bar{\varepsilon }}^{V}\right)$) and upper ($\sup\left({\bar{\varepsilon }}^{V}\right)$) values were determined using code written in the MATLAB language using calculation libraries that use the INTLAB interval and affine numbers [30]. The calculations were verified using the Monte Carlo method (MC) [31], assuming that the model parameters are random variables with a uniform distribution.

## 2. Purpose and Scope of the Research

**Figure 1:**
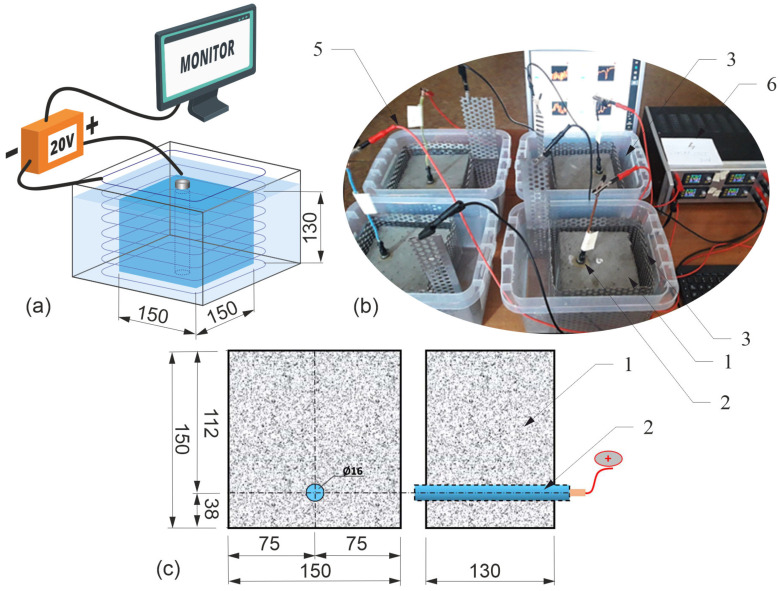
Test stand: (**a**) diagram of the test stand; (**b**) samples on the measuring stand during tests; (**c**) diagram of the test element.

As a part of laboratory tests, samples (1) with dimensions of 150 × 150 × 130 mm were made of concrete of class C50/60 (Figure 1), in which a smooth bar (2) with a diameter of Φ=16 mm and length of 150 mm made of St3SX steel (mark of the production period) was placed symmetrically at one edge with a cover thickness of $c_{\mathrm{nom}}=30\;\mathrm{mm}$ (samples marked with successive indices P8-P11), [29].

The symmetrical location of the rod guaranteed the appearance of a dominant crack in the direction of the external surface of the cover, which was beneficial due to the stability of calculations using FEM.

Strong environmental aggression was forced under laboratory conditions using the so-called accelerated corrosion test based on the electrolysis of the reinforcement. The process was regulated by applying an external voltage through a voltage-current stabilizer (6) with a recorder of operating parameters. In the electrolyzer system, the anode was a bar (2) placed inside a concrete sample (1) placed in a vessel with tap water (4), while the cathode was a perforated sheet made of weathering steel (3). The concrete pore liquid served as an electrolyte. The bar and the sheet surrounding the sample were connected with copper wires (5) with a cross-section of 2.5 ${\mathrm{mm}}^{2}$ with positive and negative poles of the external voltage source (6). The front surfaces of the bar (protruding from the concrete) were protected against the uncontrolled flow of electric current with a cover of polyester resin. 

**Figure 2:**
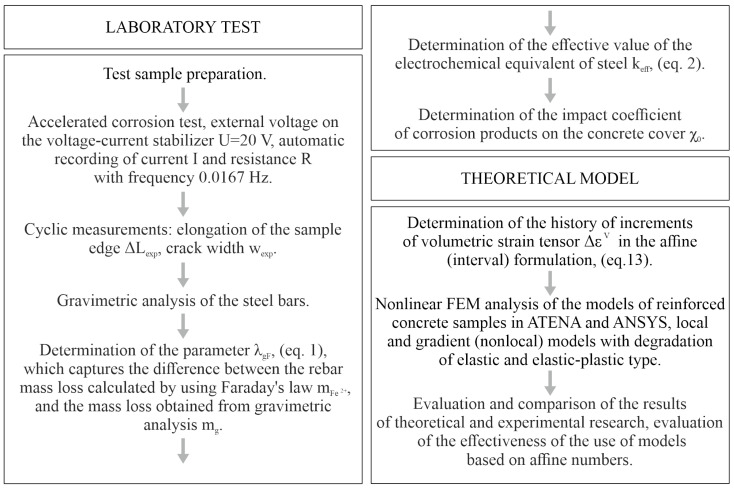
Flow chart of the experimental and theoretical tests carried out in this study.

A diagram of the experimental research carried, as well as a general outline of the theoretical research (discussed in Section 4 and Section 5) are presented in Figure 2.

During the test, the stabilizer maintained a constant voltage of 20 V in the system. Through the automatic recording of current (I) and resistance (R) with a frequency of 0.0167 Hz, the history of changes in electrical parameters during the test was monitored for each analyzed sample. The electrolysis process continued until cracks with a width of 1 mm appeared, which was accepted as sufficient to stop the process of accelerated corrosion.

**Figure 3:**
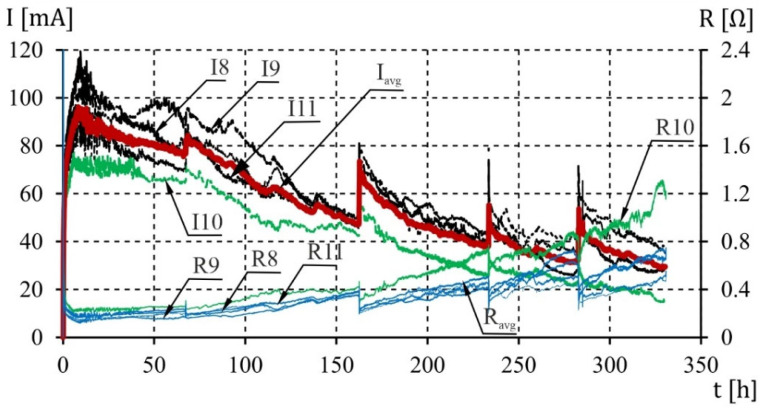
The result of the measurement of the current (I) and the electrical resistance (R) in the analyzed samples.

The course of changes in the function of the electric current and resistance of the system is shown in Figure 3.

The adopted research and measurement system, in which the test elements were completely immersed in water, compensated for deviations resulting from differences in the moisture content of the concrete structure [11]. This assumption made it possible to adopt a uniform distribution of corrosion products on the side surface of the reinforcing bar (2) subjected to the action of an external current source. Steel bars were subjected to gravimetric analysis. The loss of reinforcement mass was compared with the results obtained from theoretical analysis according to Faraday’s law. Before placing the bars in the molds, all bars were weighed (mass, $m_{g0}$). After preparing the specimens and performing the accelerated corrosion test, the cracked concrete samples were split, and the bars were removed. After derusting in phosphoric acid and mechanical cleaning, the bars were weighed again (mass, $m_{\mathrm{gt}}$), separately obtaining the mass loss ($m_{g}$) for each bar. The result of the above measurements was the determination of the $\lambda_{\mathrm{gF}}$ parameter, which captures the difference between the rod mass loss ($m_{{\mathrm{Fe}}^{2+}}$) calculated analytically using Faraday’s law and the mass loss obtained from the gravimetric analysis ($m_{g}$) [11]:

(1) $$\lambda_{\mathrm{gF}}=\frac{{\Delta m}_{e}}{m_{{\mathrm{Fe}}^{2+}}},{\;\;\;\;\;\Delta m}_{e}=m_{{\mathrm{Fe}}^{2+}}-m_{g},$$

where $m_{{\mathrm{Fe}}^{2+}}$ is the bar mass loss calculated using Faraday’s law, and $m_{g}$ is the bar mass loss determined directly from the gravimetric analysis.

The effective electrochemical equivalent of the reinforcing steel determined individually for each bar placed in the test element ($k_{\mathrm{eff}}$) was expressed as a function depending on the electrochemical equivalent ($k_{{\mathrm{Fe}}^{2+}}$ = 0.00912 g/(μA⋅year)), assuming a charge number of the reaction equal to two [11]:

(2) $$k_{\mathrm{eff}}=\left(1-\lambda_{\mathrm{gF}}\right)\cdot k_{{\mathrm{Fe}}^{2+}},$$

where $k_{{\mathrm{Fe}}^{2+}}$ is the electrochemical equivalent of iron.

The obtained test results are summarized in Table 1. The final theoretical loss of bar mass adopted for further calculations ($m_{d}$) was determined for subsequent bars based on Faraday’s law and the average value of the effective electrochemical equivalent of the reinforcing steel ($k_{\mathrm{eff},\mathrm{avg}}=0.005188\;g/\left(\mu A\cdot \mathrm{year}\right)$). The obtained average differences (${\Delta m}_{k,\mathrm{avg}}$) between the theoretically determined bar mass loss and the loss determined by gravimetric analysis did not exceed 1.5%.

**Figure 4:**
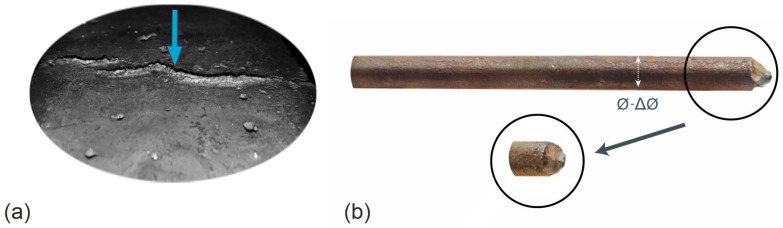
Test sample: (**a**) visible corrosion products of the reinforcement pushed through the crack in the cover; (**b**) corroded face of the reinforcing bar.

Additionally, during tests, as a result of the increase in pressure with which the corrosion products act on the concrete cover, some of the corrosion products were pushed out of the steel–concrete contact area through the crack that formed in the cover (marked with an arrow), (Figure 4a) [29]. Significant losses in reinforcing steel caused by the corrosion process occurring in the place of application of the polyester resin on the front surface of the reinforcing bar immersed in water were also observed (Figure 4b). 

The influence of the corrosion products on the values of the test results was quantitatively determined by analyzing the content of corrosion products in vessels containing tap water (electrolyte) (see Figure 1). The amount of iron compounds released during the tests was estimated based on the gravimetric analysis of the liquid in which the test samples were immersed. After the accelerated corrosion test, a volume equal to $V_{s100,n}=100\;\mathrm{mL}$ was taken from the electrolyte solution in each vessel; then, the remaining volume of the solution ($V_{s,n}$) was measured with an accuracy of 10 mL. The selected volume ($V_{s100,n}$) was filtered and dried at a temperature of more than 100 °C. The difference in the mass of the clean filter ($m_{s1}$) before and after the test, together with the dried sludge ($m_{s2}$) after the test, allowed us to determine the mass amount of corrosion products ($m_{s100}$) in a volume of $V_{s100}=100\;\mathrm{mL}$ of the solution.

(3) $$m_{s100}={\;m}_{s2}-{\;m}_{s1}$$

The mass amount of iron compounds in the entire volume of the solution ($V_{s}$) taken beyond the volume of the test element ($m_{s}$) is proportional to the mass of products contained in the volume ($V_{s100}=100\;\mathrm{mL}$). In addition, mass losses of the front surfaces of the reinforcing bars ($m_{\mathrm{out}}$) corresponding to the mass of corrosion products produced outside the steel–concrete contact area were estimated. The percentage share of corrosion products that mechanically affect the concrete cover ($m_{r}$) was determined based on the following relationship:

(4) $$m_{r\;}=\frac{\;(m_{s}-m_{\mathrm{out}})}{m_{g}}\cdot 100\%,\;\;\;\;{\;m}_{s}=\frac{{\;V}_{s}\times {\;m}_{s100}}{{\;V}_{s100}},$$

where $m_{g}$ is the actual mass of all corrosion products determined gravimetrically, $m_{s}$ is the mass of corrosion products transferred to the electrolyte solution, and $m_{\mathrm{out}}$ is the mass of products transferred to the solution from the front surfaces of the rod not directly affecting the concrete cover. A summary of the mass content of corrosion products transferred to the solution in relation to the samples analyzed in this paper is presented in Table 2.

Analysis of the test results listed in Table 2 allows one to estimate the amount of corrosion products that, as a result of the increase in the pressure of the impact of corrosion products on the cover, were pushed out of the concrete and had an impact on the propagation of a damage in the cover at the level of 50–60%. This allows for the estimation of the percentage of corrosion products affecting the cover of concrete during the electrolysis process at the level of about 40-50%. To avoid calculation problems, the average amount of corrosion products acting mechanically on concrete was assumed, which was included in the impact coefficient: $\chi_{0}=0.45$ ($P_{0}=54.7\approx 55\%$).

**Figure 5:**
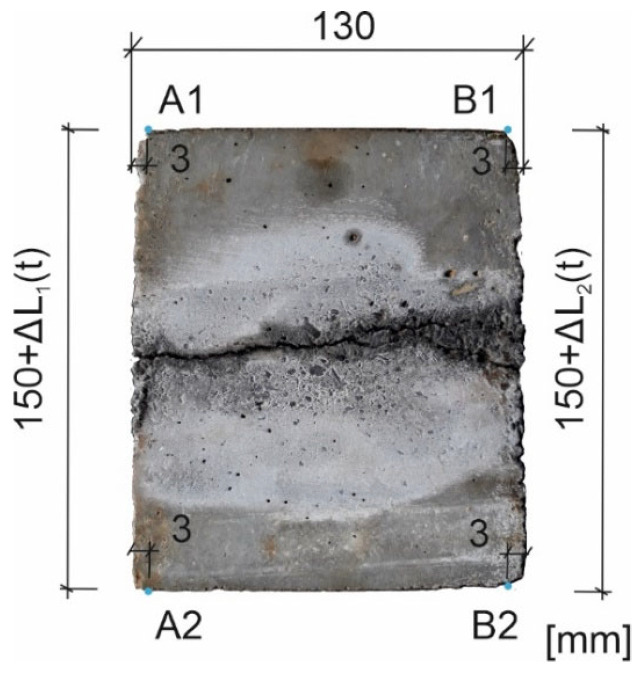
Measurements of elongation of the edge of the sample.

During the corrosion test, manual measurement of the length of the side of the scratched edge of each sample L(t) was carried out cyclically along the two extreme edges (at a distance of 3 mm from the face of the sample) with an accuracy of 0.1 mm-(Figure 5; we assume that the change in edge length is approximately equal to the average crack width). To verify this assumption, manual measurement of the width of the cracks in the element was also performed. The first (basic) measurement was performed before connecting to the electrolyzer system ($t_{1}=0\;h$), with the next measurements conducted in the following hours of the test (t≈ 43, 116, 209, 259, and 307 h.)The second measurement ($t_{2}=43\;h$) was performed in time close to the first scratch on the surface of the sample, and the last measurement was conducted at $t_{\mathrm{last}}=307\;h$. The electrolyzer was disconnected from the power supply system after $t_{\mathrm{end}}=330\;h.$

The elongation of the cracked edge of each sample at successive points of time (t) was calculated as the average value ($\Delta L_{\exp}$) determined from two extreme measurements according to the following relation:

(5) $$\Delta L_{1}=L_{1\left(t\right)}-L_{0},\;\;\;\;\;\;\;\;\;\Delta L_{2}=L_{2\left(t\right)}-L_{0},\;\;\;\;\;\Delta L_{\exp}=0.5\left(\Delta L_{1}+\Delta L_{2}\right)$$

where $\Delta L_{1}$ and $\Delta L_{2}$ are the extensions of the sample edges in two extreme positions, while $L_{1\left(t\right)}$ and $L_{2\left(t\right)}$ denote the lengths of these edges at time t.

**Figure 6:**
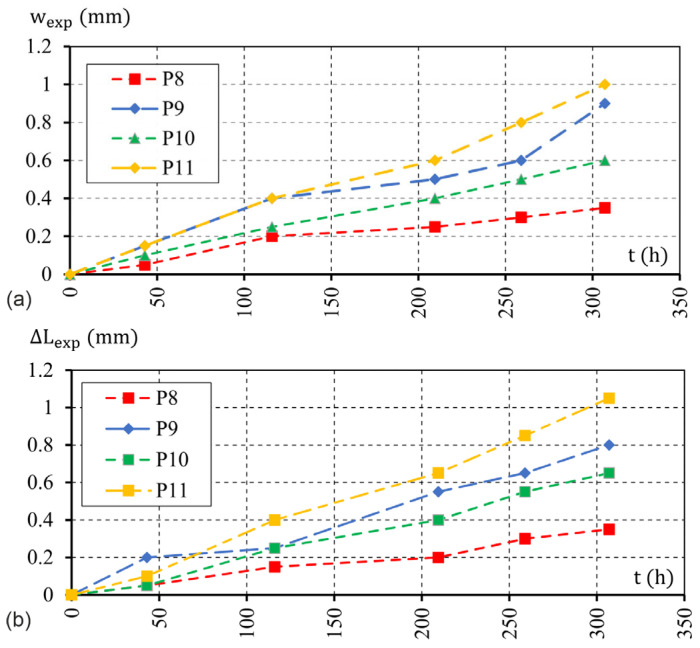
History of changes: (**a**) crack width; (**b**) elongation of the sample edge.

The history of changes in the crack width ($w_{\exp}$) and the elongation of the sample edge ($\Delta L_{\exp}$) as a function of the duration of the accelerated corrosion test are shown in Figure 6a,b, respectively [29].

As can be seen in the figure, the assumption used to identify the average length increment of the edge and the average crack width can be considered correct. It can also be seen that only the results obtained for the P9 and P11 test elements are similar. In the case of the P10 element, a smaller loss of iron ions from the reinforcement was observed, as shown in Table 1. This situation may result from the poor quality of the connection between the wire and the reinforcement bar. The value of the electrochemical equivalent of iron does not differ significantly for test items P9 and P11 (Table 1). In the case of the P8 element, a significant deviation in the displacement results obtained in the tests from the results obtained for the P9 and P11 samples can be noticed, which is caused by the appearance of additional corrosion sources located around the front surfaces of the rod as a result of strong damage of the epoxy resin protecting the front surface of the rebar.

## 3. Mathematical Model

### 3.1. Interval Tensor of Volume Strain Rate

The increase in volumetric strains of corrosion products (which is an uncertain interval value) was determined using an approach based on interval (affine) numbers using the algorithm published in [28].

According to the definition, the affine number ($\bar{X}$) can be represented as an interval number using the following relation [32,33]: ${\bar{X}}^{I}=\left[X^{-},{\;X}^{+}\right],{\;X}^{-}<X^{+},{\;X}^{-}=\inf\left({\bar{X}}^{I}\right),{\;X}^{+}=\sup\left({\bar{X}}^{I}\right)$,

(6) $$\bar{X}=X_{0}+\overset{n}{\underset{i=1}{\sum }}X_{i}{\bar{\varepsilon }}_{i}\equiv {\bar{X}}^{I}=\left[X_{0}-\mathrm{rad}\left(X\right),X_{0}+\mathrm{rad}\left(X\right)\right],\;\;\;\;\;\;\;\;\mathrm{rad}\left(X\right)=\overset{n}{\underset{i=1}{\sum }}\left|X_{i}\right|,$$

where ${\bar{X}}^{I}$ is an interval number, ${\bar{\varepsilon }}_{k}$ is a noise symbol whose value changes in the range of [−1,1], $X_{0}$ is the average value, and $X_{i}$ (i>0) is a partial deviation from the average value.

Basic algebraic operations on interval numbers are presented, among others, in [17] and a groundbreaking paper on interval numbers [23].

(7) $${\bar{X}}^{I}\pm {\bar{Y}}^{I}=\left[X^{-}\pm Y^{-},X^{+}\pm Y^{+}\right],$$

(8) $${\bar{X}}^{I}\cdot {\bar{Y}}^{I}=[\min\left(X^{-}Y^{-},X^{-}Y^{+},X^{+}Y^{-},X^{+}Y^{+},\max\left(X^{-}Y^{-},X^{-}Y^{+},X^{+}Y^{-},X^{+}Y^{+}\right)\right],$$

(9) $${\bar{X}}^{I}/{\bar{Y}}^{I}=\left[X^{-},X^{+}]\cdot [1/Y^{+},1/Y^{-}\right].$$

Similar operations with respect to affine numbers can be found in the other texts [23,26,32,33,34].

(10) $$\bar{X}\pm \bar{Y}=X_{0}\pm Y_{0}+\sum_{i=1}^{n}\left(X_{i}\pm Y_{i}\right){\bar{\varepsilon }}_{i},$$

(11) $$\bar{X}\cdot \bar{Y}=X_{0}\cdot Y_{0}+\sum_{i=1}^{n}\left(X_{0}Y_{i}+X_{i}Y_{0}\right){\bar{\varepsilon }}_{i}+\sum_{i=1}^{n}X_{i}{\bar{\varepsilon }}_{i}\cdot \sum_{i=1}^{n}Y_{i}{\bar{\varepsilon }}_{i},$$

(12) $$\bar{X}/\bar{Y}=\bar{X}\cdot {\left(\bar{Y}\right)}^{-1}≅\bar{X}\bar{Z}.$$

The method of determining the function $\bar{Z}=1/\bar{Y}$ in Equation (12) is presented, among others, in [30,35] using Chebyshev approximation. In the case of libraries defined in the INTLAB environment that were used to perform the calculations, the result of the multiplication is considered common between the interval and affine arithmetic operations. The result of multiplication can never be worse than in simple interval arithmetic [30].

### 3.2. Use of Affine Numbers to Describe the Effect of Corrosion Products on Concrete

The impact of corrosion products on concrete in the affine approach can be described using the interval tensor of the rate of volumetric strain, which, in the modified interval form, becomes [11,28,36]

(13) $${\dot{\bar{\varepsilon }}}_{\alpha \beta }^{V}={\dot{\bar{\varepsilon }}}^{V}\delta_{\alpha \beta }=\frac{\left(1-\beta \right){\dot{\bar{V}}}_{\mathrm{ekw}}}{{\eta V}_{0}}\delta_{\alpha \beta },\;\;\;\;\;\;\;\eta =3\beta +2\left(1-\beta \right),\;\alpha ,\beta =1,2.$$

where β is a function of the increase in the interaction of corrosion products with the cover due to the sealing of the transition layer, is the interval velocity of the equivalent volume, η is a function that includes the sealing of the products in pores and cracks of the cover ($\eta =2\;\mathrm{for}\;t>t_{\mathrm{cr},0})$, $t_{\mathrm{cr},0}$ is critical time and $\delta_{\alpha \beta }$ is the Kronecker delta.

When describing a problem with interval numbers, the parameters of the model are defined by intervals. The following values are treated as interval parameters: $\bar{\alpha },\bar{\vartheta }$, describing the composition of corrosion products [36]; ${\bar{\gamma }}_{\mathrm{wp}}$, defining the porosity of the transition zone; ${\bar{w}}_{\mathrm{wp}}$, defining the width of the transition zone; and ${\bar{k}}_{\mathrm{eff}}$, representing the effective electrochemical equivalent of iron.

Furthermore, derived quantities such as pore space volume (${\bar{V}}_{\mathrm{por}}$), transition zone volume (${\bar{V}}_{\mathrm{wp}}$) [5], and an effective and equivalent volume of corrosion products, become interval values:

(14) $${\bar{V}}_{\mathrm{por}}={\bar{\varepsilon }}_{\mathrm{wp}}{\bar{V}}_{\mathrm{wp}}\Rightarrow {\bar{V}}_{\mathrm{por}}=\left[V_{\mathrm{por}}^{-},V_{\mathrm{por}}^{+}\right],$$

(15) $${\bar{V}}_{\mathrm{wp}}={\bar{w}}_{\mathrm{wp}}\pi D\Rightarrow {\bar{V}}_{\mathrm{wp}}=\left[V_{\mathrm{wp}}^{-},V_{\mathrm{wp}}^{+}\right]$$

(16) $${\dot{\bar{V}}}_{\mathrm{eff}}=\left(1-\beta \right)\bar{\omega }{\dot{\bar{V}}}_{\mathrm{ekw}},\;\;\;\;\;{\dot{\bar{V}}}_{\mathrm{ekw}}={\bar{V}}_{R}-{\bar{V}}_{\mathrm{Fe}}{}_{2^{+}},\;\;\;\;\;{\bar{V}}_{\mathrm{Fe}}{}_{2^{+}}={\bar{V}}_{\mathrm{Fe}}{}_{2^{+}}\left(I\right),$$

(17) $${\dot{\bar{V}}}_{\mathrm{ekw}}=\frac{\left({\bar{\alpha }}^{-1}\bar{\vartheta }-1\right){\bar{k}}_{\mathrm{comp}}\bar{I}}{\rho_{{\mathrm{Fe}}^{2+}\;}},\;\;\;\;\;{\bar{k}}_{\mathrm{comp}}={\bar{\chi }\bar{k}}_{\mathrm{eff}},$$

where D is the bar diameter, ${\dot{\bar{V}}}_{\mathrm{eff}}$ is the rate of the effective volume of corrosion products, ${\dot{\bar{V}}}_{R}$ is the rate of volume of reinforcement corrosion products, ${\dot{\bar{V}}}_{{\mathrm{Fe}}^{2+}}$ is the rate of volume of the iron ions corresponding to the corrosion pit, $\bar{\omega }$ is a parameter that shows the influence of uncertainty on the time and the mechanical impact of corrosion products on concrete, and $\bar{\chi }$ is a parameter describing the amount of corrosion products that interact effectively with the cover.

Parameter $\bar{\omega }$ shows the influence of uncertainty on the time of initiation and the method of mechanical impact of corrosion products on concrete. The parameter describes three independent computational situations [28]: (a) no interaction ($t<t_{0}^{-},{\;V}_{\mathrm{ekw}}^{+}<V_{\mathrm{por}}^{-}$, $\bar{\omega }=\left[0,0\right]$), (b) unconditional interaction between corrosion products and the cover ($t>t_{0}^{+},{\;V}_{\mathrm{por}}^{+}<V_{\mathrm{ekw}}^{-}$, $\bar{\omega }=\left[1,1\right]$), and (c) an intermediate situation in which both cases are possible ($t_{0}^{-}\leq t\leq t_{0}^{+}$, $\bar{\omega }=\left[0.1\right]$). The interval parameter ($\bar{\omega }$) is characterized by the following relationship:

(18) $$\bar{\omega }=\{\begin{array}{l} \left[0.0\right],\;\;\;\;\;t<t_{0}^{-},{\;\;\;\;\;V}_{\mathrm{ekw}}^{+}<V_{\mathrm{por}}^{-},\\ \left[0.1\right],{\;\;\;\;\;t}_{0}^{-}\leq t\leq t_{0}^{+},{\;\;\;\;\;V}_{\mathrm{por}}^{-}\leq V_{\mathrm{ekw}}^{+}\;\wedge {\;V}_{\mathrm{ekw}}^{-}\leq V_{\mathrm{por}}^{+},\\ \left[1.1\right],\;\;\;\;\;t>t_{0}^{+},{\;\;\;\;\;V}_{\mathrm{por}}^{+}<V_{\mathrm{ekw}}^{-}, \end{array}$$

where $t_{0}^{-}$ and $t_{0}^{+}$ are the lower and upper limits of the initiation time of the reinforcement corrosion process, respectively; $V_{\mathrm{por}}^{-}$ and $V_{\mathrm{por}}^{-}$ are the lower and upper limits of the pore space volume, respectively; and $V_{\mathrm{ekw}}^{-}$ and $V_{\mathrm{ekw}}^{+}$ are the lower and upper limits of the equivalent volume, respectively.

The function β describes the three phases of the interaction between the corrosion products and the cover. In order to avoid excessive computational complications, in the interval analysis, it was assumed that this parameter is deterministic and is described by dependencies [11,28,37]:

(19) $$\beta =\{\begin{array}{l} 1,\;\;\;\;\;t<t_{0}\approx t_{0}^{-},{\;\;\;\;\;V}_{\mathrm{ekw}}^{+}<V_{\mathrm{por}}^{-},\\ (t_{\mathrm{cr}}-t)/\left(t_{\mathrm{cr}}-t_{0}\right),{\;\;\;\;\;t}_{0}\approx t_{0}^{-}\leq t\leq t_{\mathrm{cr},0},{\;\;\;\;\;V}_{\mathrm{por}}^{-}\leq V_{\mathrm{ekw}}^{+}\wedge {\;\;\;\;\;V}_{\mathrm{ekw}}^{-}\leq V_{\mathrm{cr}}\;\\ 0,\;\;\;\;\;t>t_{\mathrm{cr},0},{\;\;\;\;\;V}_{\mathrm{ekw}}^{-}>V_{\mathrm{cr}}. \end{array}.$$

- Phase I: For $t\leq t_{0}^{-}$, ${\bar{t}}_{0}=\left[t_{0}^{-},t_{0}^{+}\right]$, β=1, no interaction of corrosion products with concrete cover (it was assumed that $t_{0}^{-}\approx t_{0}^{+}\approx t_{0}$, ${\Delta t}_{0}=t_{0}^{+}-t_{0}^{-}\ll {\Delta t}_{\mathrm{cr}}=t_{\mathrm{cr}}-t_{0}$);
- Phase II: For $t_{0}^{-}=t_{0}\leq t\leq t_{\mathrm{cr},0}$, β∈(0,1), with a gradual increase in the impact of corrosion products on the concrete cover; microcracks in the cover structure join together, and pore spaces are filled;
- Phase III: For $t>t_{\mathrm{cr},0}$ (β=0), corrosion products have the maximum effect on concrete cover ${\dot{\bar{V}}}_{\mathrm{eff}}={\dot{\bar{V}}}_{\mathrm{ekw}}$.

### 3.3. Material Models

#### Introduction

The material models defined in the ANSYS program were used in the calculations. For verification, the calculations were compared with the results available for the model implemented in ATENA software. Calculations were carried out using:

- An elastic–plastic concrete model with a Menetrey–Willam surface with hardening/softening in compression and tension (HSD2 model): The model is implemented in the ANSYS program [38], is independent of the FEM mesh, and is dependent on the fracture energy. Details on the MW model and its computer implementation can be found in [39,40].
- An elastic–plastic concrete model with cracking and Menetrey–Willam and Rankine surfaces in the tension region (CC3DNonLinCementitious2 model): The model is implemented in ATENA software, is independent of the FEM mesh, and is dependent on the fracture energy. Details on the MWR model, its implementation, formulation of FEM equations, and algorithms of the ATENA program can be found in [41,42].
- An EDM concrete microplane model with degradation: The model is independent of the FEM mesh and it is gradient-regularized [38,43].
- Contact was described by considering the Coulomb friction model.
- For steel, a classic perfectly elastic–plastic model without hardening was used.

## 4. Material Parameters and Analysis of Calculation Example

Calculations of the edge displacements of the test elements (ANSYS and ATENA) were performed, in which the uncertainty of the model parameters was assumed. The calculations were performed using the finite element method (FEM). The uncertainty of the model parameters was shown by declaring numerical ranges in accordance with the approach proposed in [28]. As a way of estimation, the calculations were verified using the MC method (500 draws) for a uniform distribution of random variables [31]. The loadings were the lower ($\inf\left({\Delta \bar{\varepsilon }}_{\alpha \beta }^{v}\right)$) and upper (sup$\left({\Delta \bar{\varepsilon }}_{\alpha \beta }^{v}\right)$) limits of the coordinates of the interval tensor of volumetric strains (in the case of the MC method, the limit values of a random variable with linear distribution). The purpose of the calculations was to determine the limit increments of displacements of the sample edges as a function of experimental time. The displacement increments were assumed to be approximately equal to the average crack widths.

It was assumed that the corrosion products formed on the surface of the reinforcement are a mixture of hydroxides ($\mathrm{Fe}{\left(\mathrm{OH}\right)}_{2}$ and $\mathrm{Fe}{\left(\mathrm{OH}\right)}_{3}$). Average parameters characterizing the composition of the corrosion products ($\alpha_{0}$ and $\vartheta_{0}$) were adopted in accordance with [36]. To simplify the interval computational algorithm, it was assumed that the critical time, the value of which is, by definition, lower than the cover cracking time, is a deterministic parameter ($t_{\mathrm{cr},0}=43\;h$) equal to the cracking time of the test element.

As interval parameters, the quantities describing the chemical composition of corrosion products ($\bar{\alpha }$ and $\bar{\vartheta }$), porosity (${\bar{\varepsilon }}_{\mathrm{wp}}$), the width of the transition layer (${\bar{w}}_{\mathrm{wp}}$), the multiplier of the corrosion products impact intensity ($\bar{\chi }$), and the electrochemical equivalent of iron (${\bar{k}}_{\mathrm{eff}}$) were adopted. It was assumed that the specific density of iron ions is a deterministic parameter ($\varrho_{{\mathrm{Fe}}^{2+}}=7850\;\mathrm{kg}/m^{3}$). Deviations of the model parameters ($\bar{X}$) from the mean value were calculated according to the following relationship:

(20) $$\bar{X}={\bar{X}}_{0}-\mathrm{rad}\left(\bar{X}\right),{\bar{X}}_{0}+\mathrm{rad}\left(\bar{X}\right),\;\;\;\;\;\mathrm{rad}\left(\bar{X}\right)=\frac{1}{2}\left(\inf\left(\bar{X}\right)-\sup\left(\bar{X}\right)\right)=\frac{1}{2}\mu_{\%}{\bar{X}}_{0}$$

where $\mu_{\%}$ is the percentage deviation, $\bar{X}$ is the range, ${\bar{X}}_{0}$ is the mean value, $\mathrm{rad}\left(\bar{X}\right)$ is the deviation from the mean value, $\sup\left(\bar{X}\right)$ is the upper bound of the interval, $\mathrm{and}\;\inf\left(\bar{X}\right)$ is the lower bound of the interval.

Mean values, as well as upper and lower limits of the range numbers, are presented in Table 3, assuming the variability of the $\mu_{\%}$ parameter at the level of 10% and 20%. In the case of Monte Carlo calculations, the declared numerical ranges corresponded to uniformly distributed random variables. The interval defining the critical time was only considered for the MC analysis. In the case of calculations using affine (interval) numbers, the mean value of the critical time ($t_{\mathrm{cr},0}$) was used, which allowed for the simplification of the algorithm in accordance with the comments presented in Section 3.2.

**Figure 7:**
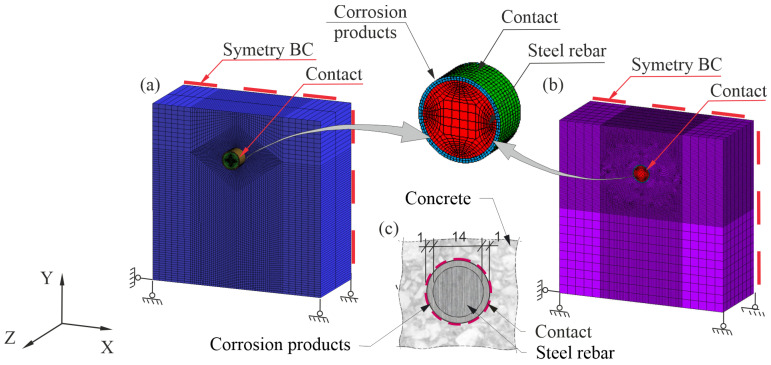
FEM models of reinforced concrete samples analyzed in this paper: (**a**) model built in the ATENA program; (**b**) model built in the ANSYS program; (**c**) steel–concrete contact region.

In order to perform computer analyses, a finite element mesh was generated. Both models were realized as symmetrical in a plane perpendicular to the element axis. The models and means of support implemented in both ATENA and ANSYS are presented in Figure 7.

**Figure 8:**
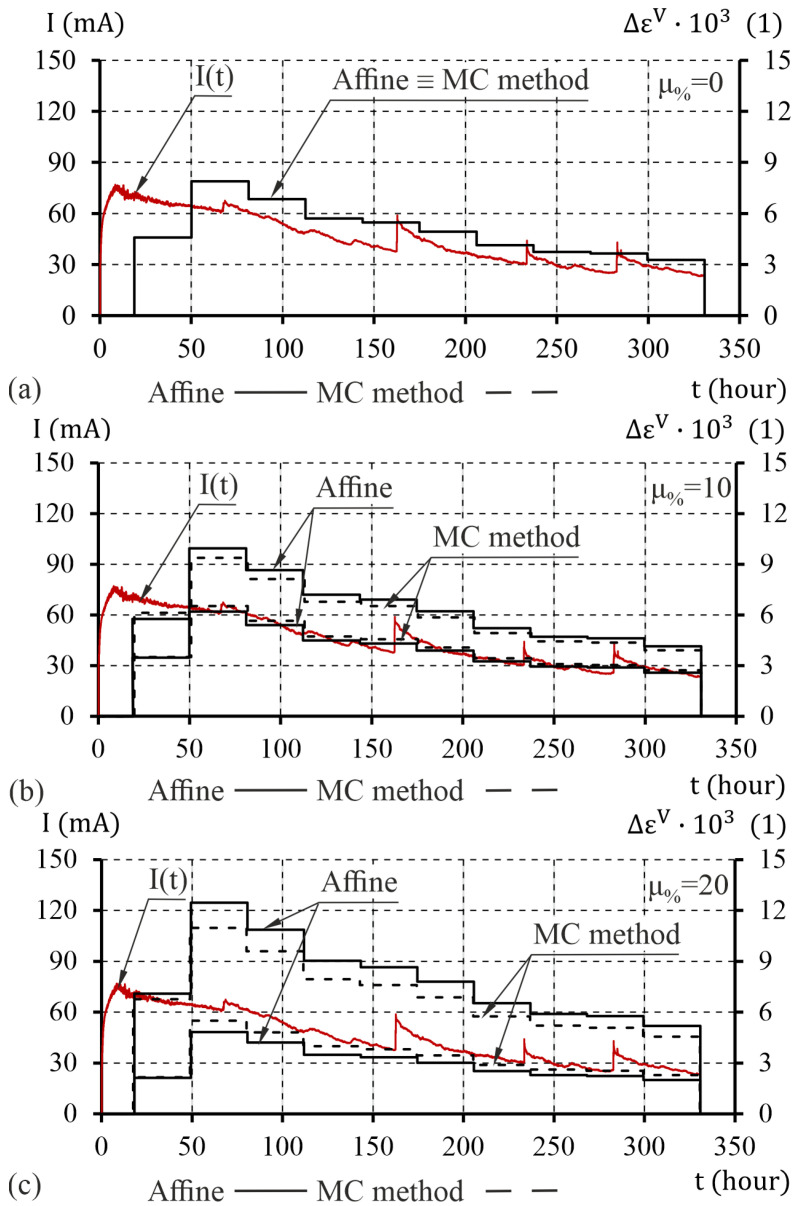
Distribution of the increments of volumetric strain tensor caused by corrosion products (${\Delta \varepsilon }_{\mathrm{kor}}$): (**a**) 0% deviation, (**b**) 10% deviation, and (**c**) 20% deviation.

In the ATENA software (Figure 7a) standard eight-node solid elements were used. In ANSYS software (Figure 7b), the material was modeled with solid185 and cpt215 elements, while contact interactions were modeled with conta174 and targe170 elements [38]. Due to the use of the EDM gradient model, the mesh was densified in the area where cracks were expected. In the plane perpendicular to the rod axis, finite elements with a dimension of 1 mm were assumed. In the longitudinal direction, the model was divided into eight finite elements (two elements in ATENA software). In the ANSYS program, this type of mesh was also used in calculations using the elastic–plastic model. The total increment of volumetric strains caused by the deposition of corrosion products on the side surface of the reinforcing bar was carried out in 10 calculation steps. Loads were applied in the form of increments of the tensor of volumetric strain to a substitution ring located around the reinforcing bar in a plane perpendicular to the axis of the reinforcement (Figure 7c). Due to the interval nature of the tensor of increment of volumetric strain, the evaluation of the impact of uncertainty on the degradation of the concrete cover was analyzed for its lower ($\inf\left({\Delta \bar{\varepsilon }}^{V}\right)$) and upper ($\sup\left({\Delta \bar{\varepsilon }}^{V}\right)$) limit values. The courses of changes of the tensor of increments of volumetric strain for the assumed uncertainties ($\mu_{\%}=0$, $\mu_{\%}=10\%$, and $\mu_{\%}=20\%$) are shown in Figure 8. The calculations were performed using three different material models of concrete cover. The elastic parameters common to the models are listed in Table 4 and Table 5.

The inelastic parameters are listed in tables depending on the material model and the software: (a) elastic–plastic model of material with cracking (MWR, CC3DnonLinCementitious2), ATENA program, Table 6; (b) elastic–plastic material model with strengthening and softening (MW with HSD2), ANSYS program, Table 7; (c) nonlocal gradient-formulated elastic microplane with degradation (EDM), ANSYS program, Table 8; [39,41,43]. The interactions of steel and concrete were described by introducing a rigid contact model, which was adopted for both the model built in the ATENA program and the models defined in the ANSYS program (Table 9).

## 5. Discussion of Calculation Results

**Figure 9:**
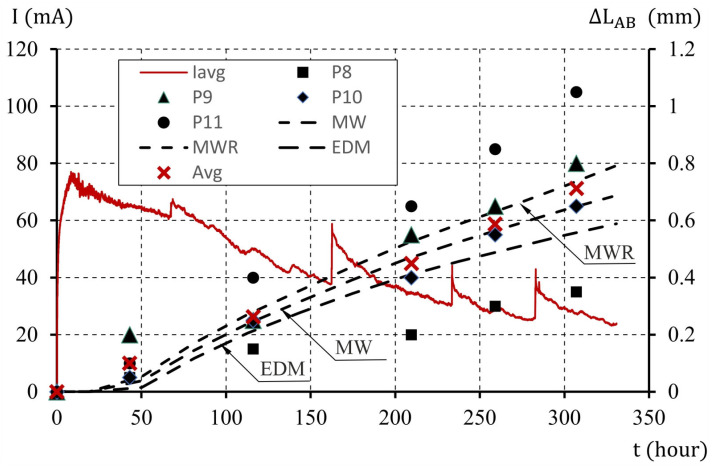
The course of changes in the elongation of the sample edge (the average width of the crack), with an interaction parameter of $\chi_{0}=0.45$ and model parameter uncertainty of $\mu_{\%}=0\%$.

As a result of the calculations, the values of the relative displacements of points A and B (${\Delta L}_{\mathrm{AB}}$) in the analyzed test element (average crack width) were determined. Graphical images showing the elongations of the edges were generated, taking into account uncertainties of the model parameters ($\mu_{\%}$) of 0, 10, and 20%. The results obtained with model parameter uncertainty of $\mu_{\%}=0\%$ are presented in Figure 9. The results oscillate around the average values of displacement increments obtained for the examined test elements. The percentage deviations of the results of computer tests of the analyzed MWR and MW models are characterized by high compliance with the obtained calculation results, with percentage discrepancy between the test results of approximately 4% and 8%, respectively. The deviation from the mean value obtained for the EDM gradient model was greater (approximately 21%; Table 10).

**Figure 10:**
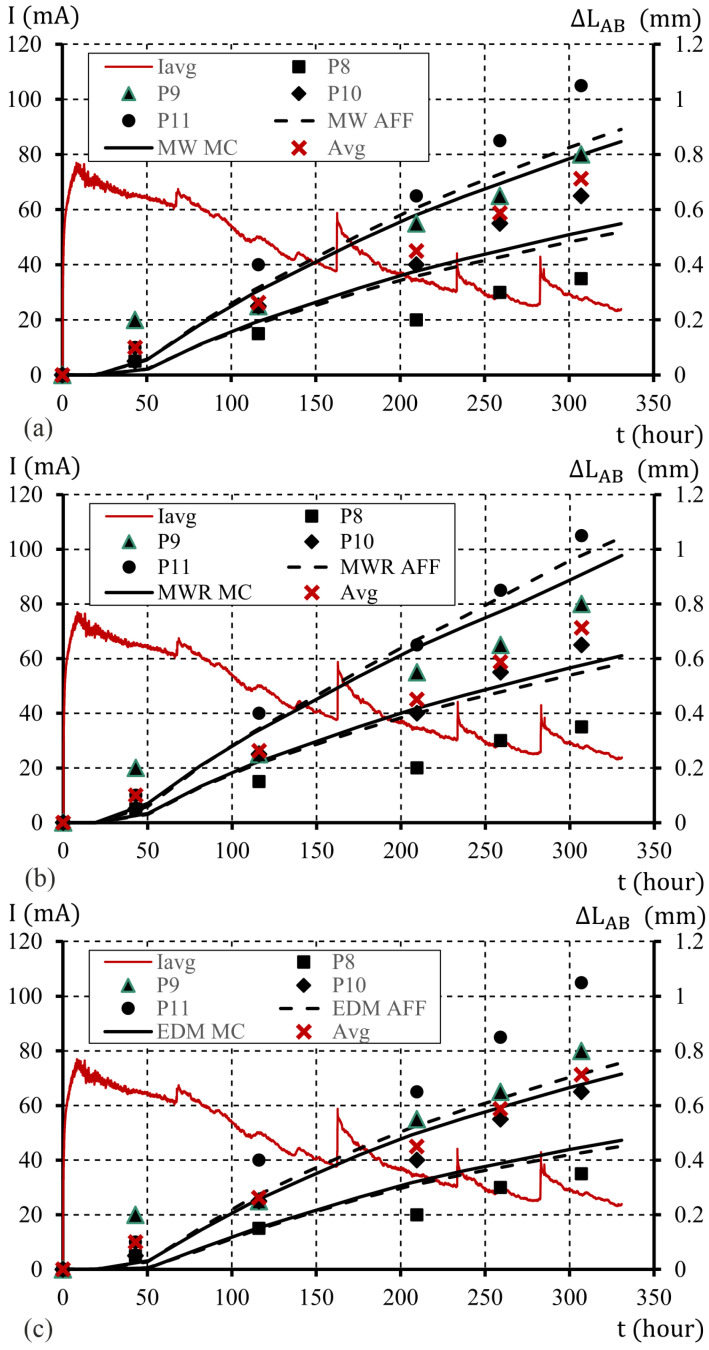
The course of changes in the elongation of the sample edge (the average width of the crack), with an interaction parameter of $\chi_{0}=0.45\;$, model parameter uncertainty $\mu_{\%}=10\%$, MC and Affine approach, models: (**a**) MW; (**b**) MWR; (**c**) EDM (description in text).

**Figure 11:**
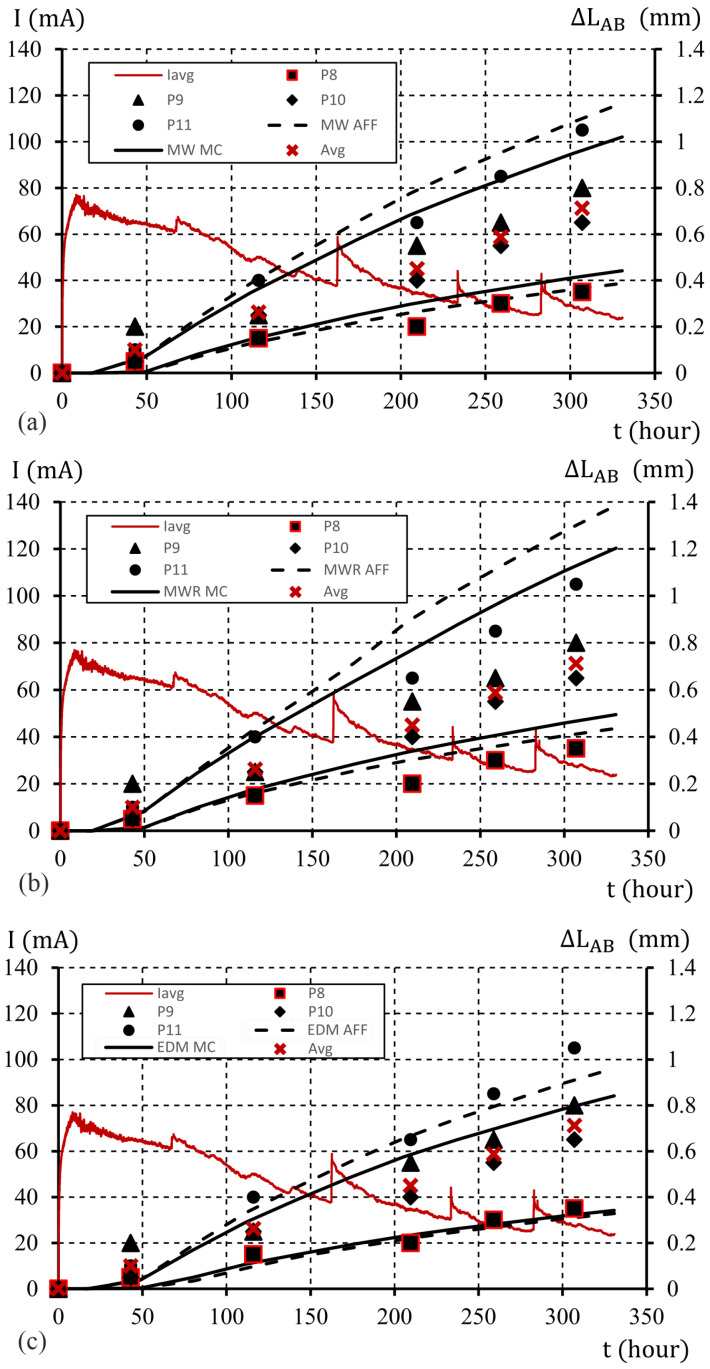
T he course of changes in the elongation of the sample edge (the average width of the crack), with an interaction parameter of $\chi_{0}=0.45\;$, model parameter uncertainty $\mu_{\%}=20\%$, MC and Affine approach, models: (**a**) MW; (**b**) MWR; (**c**) EDM (description in text).

A graphical image of the sample edge displacements for the assumed uncertainties at the level of $\mu_{\%}=10\%$ is shown in Figure 10. The curves obtained from the limit displacement values include the results of the measurements obtained for test elements P9 and P10. In the case of calculations using the MWR model (ATENA program), the obtained boundary curves also include the results of experimental tests obtained for the P11 element. An assumption of $\mu_{\%}=20\%$ allowed us to achieve limit curves of displacements that include all the obtained results of experimental tests, as shown in Figure 11.

It should be noted that the results obtained for the affine approach and the MC method are very similar, with deviations close to 10%. With larger deviations of the model parameters, the results start to differ slightly, especially in relation to those obtained for the upper end of the solution interval ($\sup\left(\bar{X}\right)$). In the case of a model based on the MWR model, the deviations of the calculation result curves ($\inf\left(\bar{X}\right)$ and $\sup\left(\bar{X}\right)$) using the affine approach and MC start to differ from each other in a significant way. This is most likely due to problems with the convergence of the solution in the ATENA software for increments of total volumetric strains. Furthermore, based on the course of changes of displacements presented in Figure 9, Figure 10 and Figure 11, the limit values of time ($t_{\min}=\inf\left(\bar{t}\right)$ and $t_{\max}=\sup\left(\bar{t}\right)$) at which the limit width of the opening of the crack can be reached are presented in Table 11. The estimated limit value ($w_{\mathrm{avg}}\approx {\Delta L}_{\mathrm{AB}}=0.4\;\mathrm{mm}$) was accepted. Time interval ($\bar{t}$) in which these cracks may occur, assuming $\mu_{\%}=10\%$, varies in the range of $\langle \Delta \bar{t}=25.2,40.0\rangle$ percent of the total duration of the process. In the case of $\mu_{\%}=20\%$, the time interval of reaching the critical crack width change is in the range of $\langle \Delta \bar{t}=60.8,70.3\rangle$ as a percentage of the total duration of the process.

**Figure 12:**
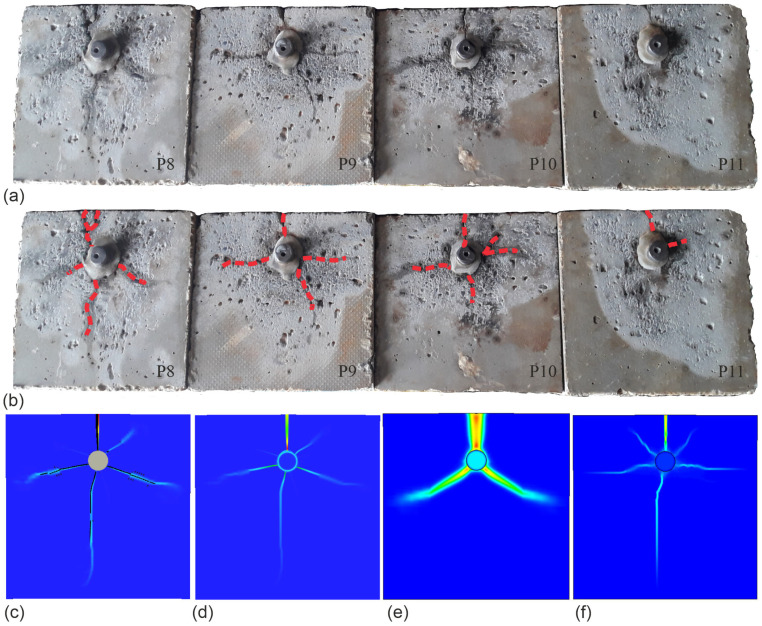
Observed and predicted failure crack patterns of the sample (χ=0.45; time t=330 h): (**a**) observed failure crack patterns; (**b**) applied crack pattern; (**c**) map of cracks obtained in the MWR model; (**d**) map of total principal tensile strains in the MW model; (**e**) map of total principal tensile strains in the EDM model (**f**); map of total principal tensile strains in the MW model.

To compare damages, the cracks in the real samples (P8–P11) and the total principal tensile strain values obtained for the FEM simulation after $t_{\mathrm{end}}=330\;h$ of the accelerated reinforcement corrosion process were analyzed. The crack patterns obtained after examining the actual test elements are shown in Figure 12a, while in Figure 12b shows the courses of changes of crack evolution in subsequent samples marked by red doted lines. To verify the experimental tests, damage maps (cracks and total principal tensile strains) obtained by computer simulations are shown in Figure 12c–f. The following maps are presented: a map of cracks obtained in the MWR model (Figure 12c), a map of total principal tensile strains in the MWR model (Figure 12d), a map of total principal strains in the EDM model (Figure 12e), and a map of total principal tensile strains in the MW model (Figure 12f).

The computer-generated maps of total principal tensile strains and cracks are similar to the damage images captured on the photographed test elements, in particular with regard to the calculations obtained for the total principal tensile strains in the MW model (Figure 12f) and both crack distribution and total principal tensile strains for the MWR model (Figure 12c,d). In the case of the results obtained for the gradient model, the graphical images of total principal tensile strains differ in shape and position from the results of the experimental tests.

**Figure 13:**
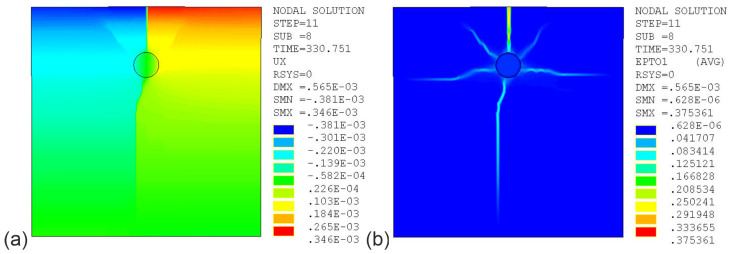
Maps of tensor and scalar fields for the MW model with HSD2 (ANSYS) (χ=0.45, time t=330 h): (**a**) displacements ($u_{x}$); (**b**) total principal tensile strain ($\varepsilon_{I}$).

**Figure 14:**
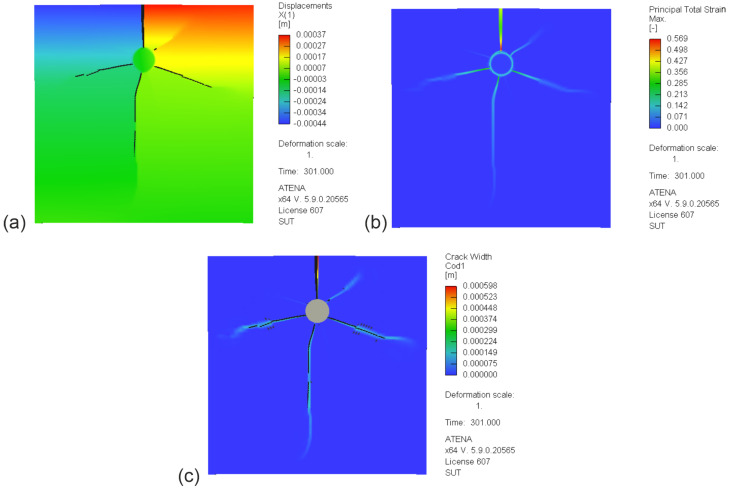
Maps of tensor and scalar fields for the MWR model (ATENA) (χ=0.45, time t=330 h): (**a**) displacements ($u_{x}$); (**b**) total principal tensile strain ($\varepsilon_{I}$); (**c**) cracks in the test element.

**Figure 15:**
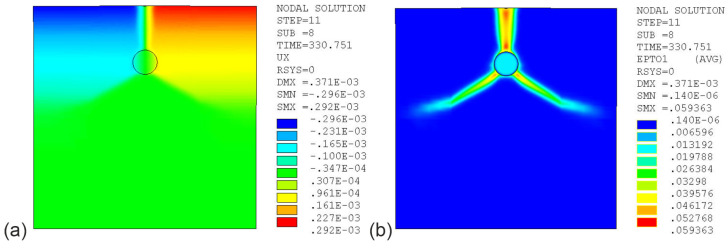
Maps of tensor and scalar fields for the EDM model (ANSYS) (χ=0.45, time t=330 h): (**a**) displacements ($u_{x}$); (**b**) total principal tensile strain ($\varepsilon_{I}$).

The results of simulation tests, maps of displacements ($u_{x}$) in the X direction, cracks in the test element in the case of ATENA software, and total principal tensile strains at t =330 h depending on the material model and software are shown in Figure 13, Figure 14 and Figure 15, with model parameter uncertainty of $\mu_{\%}=0\%$ and a corrosion interaction parameter of χ=0.45.

Figure 13a,b show graphical images of displacement fields ($u_{x}$) and principal tensile strain ($\varepsilon_{I}$), respectively. Graphical images were obtained using ANSYS software for the elastic–plastic model MW with HSD2. The displacements in this model are objective and independent of the FEM mesh. In terms of strains, the model is not objective; strains are localized in a single finite element. As previously mentioned, when analyzing the graphical images obtained in Figure 12, the maps objectively reproduce the image of cracks in the test elements.

Figure 14 presents the results of calculations obtained with ATENA software for the two surface MWR models. The graphical images present the distribution of displacements ($u_{x}$, Figure 14a), the distribution of principal tensile strain ($\varepsilon_{I}$, Figure 14b), and the distribution of the cracks in the test element (Figure 14c). The maps of the obtained calculation results presented in Figure 13 and Figure 14 are similar in terms of both the obtained displacement and strain values. It is worth emphasizing once again that the two local elastic–plastic models are formulated similarly from a theoretical point of view.

Figure 15 shows graphical images obtained for the last series of model studies. The calculations were performed using a gradient EDM model. The following graphical images are presented: the displacement distribution ($u_{x}$, Figure 15a) and the distribution of principal tensile strain (Figure 15b). The obtained graphical images of the total principal tensile strain differ from the image of the crack’s distribution obtained in the experimental study (Figure 12). However, it should be emphasized that the total principal tensile strain in the EDM model representing the location of cracks is objective in terms of strain value and reflects the actual strain results in the test sample (in the major crack). It can also be noted that the displacement maps shown in Figure 13, Figure 14 and Figure 15 are consistent with the results of elongation (${\Delta L}_{\mathrm{AB}}$) presented in Figure 9; despite the discrepancies in the obtained research results, they should be considered a good approximation of the experiment.

## 6. Conclusions

Analysis of the calculation results indicates the high efficiency of the proposed approach in predicting the propagation of damage in the reinforced concrete elements analyzed during accelerated corrosion tests. The results obtained using the MC method and the interval approach based on affine numbers and the presented algorithm of the procedure achieve similar results in the analyzed uncertainty range. With larger (above $\mu_{\%}=10\%$) model parameter uncertainties, a slight increase in discrepancies can be seen between the results obtained using the affine approach and those obtained using the MC method. The proposed approach to estimating the influence of uncertainty on the time of damage propagation in the cover can be easily extended to problems in which we deal with natural corrosion of the reinforcement. The predicted time of possible damage to the elements at which the limit width of the cracks may or may not be reached increases significantly with increasing uncertainty of the model parameters. However, taking into account that a difference in the time of occurrence of a crack width equal to 0.4 mm (equal to the increase in the length of the element) for the extreme calculation results obtained for the MWR and EDM models (Figure 9) is about 40 h (which is about 13% of the total process time), the obtained results should be considered a reliable assessment of the time interval in which damage to the element may occur.

Analyzing the results of computer research, it should be noted that with respect to the issues in which real corrosion processes and the assessment of the prospects for damage development in a degrading reinforced concrete element take place, there may be very significant discrepancies between the observed state and that which may take place as the corrosion process develops. Even with relatively small differences in forecasts regarding the conditions under which the reinforcement corrosion process occurs, the obtained results may differ significantly.

The calculations clearly show that the proposed approach effectively reflects the impact of uncertainty on the obtained discrepancies in research results. These results are similar to those obtained using the MC method and assuming a linear distribution of random variables, as shown by tests for uncertainties oscillating within limits of 20%. In addition, it should be stated that the proposed approach seems to be a forward-looking one, especially when attempting to narrow down the obtained results, e.g., by introducing fuzzy numbers and a probabilistic approach.

Further development of the research presented in this paper may also be undertaken within the scope of implementation of the presented model for full-size structural elements operating under real conditions of an aggressive environment.

## Figures and Tables

**Table 1:** Experimental results of mass loss of the rebars and the coefficients of the electrochemical equivalent of steel.

No	$m_{g0}$ (g)	$m_{gt}$ (g)	$m_{g}$ (g)	$m_{Fe^{2+}}$ (g)	$\Delta m_{e}$ (g)	$\lambda_{gF}$	$k_{eff}\cdot 10^{3}$ (g/μA · Year)	$m_{d}$ (g)	$\Delta m_{k}$ (%)
$P_{8}$	228.53	215.93	12.601	20.702	8.10	0.391	0.005551	12.480	1.0
$P_{9}$	224.39	212.02	12.369	21.156	8.79	0.415	0.005332	12.754	3.0
$P_{10}$	227.95	220.98	6.963	14.913	7.95	0.533	0.004258	6.963	0.0
$P_{11}$	227.45	215.09	12.354	20.082	7.73	0.385	0.005610	12.106	2.0
$P_{0}$	227.079	216.007	11.072	19.213	8.14	0.424	0.005188	11.076	1.5

**Table 2:** Measurement results of the mass of corrosion products transferred into the solution.

Nr	$m_{s1}$ (g)	$m_{s2}$ (g)	$m_{s100}$ (g)	$V_{s}\;$ (mL)	$m_{s}$ (g)	$m_{out}$ (g)	$m_{r\;}$ (%)
$P_{8}$	1.232	1.5	0.268	3360	9.005	1.539	59.2
$P_{9}$	1.213	1.472	0.259	3600	9.324	1.911	59.9
$P_{10}$	1.206	1.355	0.149	3720	5.543	2.096	49.5
$P_{11}$	1.168	1.366	0.198	3700	7.326	1.162	49.9
$P_{0}$	1.205	1.423	0.219	3595	7.799	1.677	54.7

**Table 3:** List of mean values and lower ($X^{-}=\inf\left(\bar{X}\right)$) and upper ($X^{+}=\sup\left(\bar{X}\right)$) ranges describing the parameters of the model (random variables with uniform distribution in the case of the MC method).

Calculation Parameter	$X_{0}$	$inf\left(\bar{X}\right)$	$sup\left(\bar{X}\right)$	$inf\left({\bar{X}}_{20\%}\right)$	$sup\left({\bar{X}}_{20\%}\right)$
		$\mu_{\%}=10\%$		$\mu_{\%}=20\%$	
Electrochemical equivalent of steel, ${\bar{k}}_{\mathrm{eff}}\cdot 10^{-3}$ (g/μArok)	5.188	4.9286	5.4474	4.6692	5.7068
Parameter $\bar{\chi }$ (1)	0.45	0.4275	0.4725	0.405	0.495
Parameter $\bar{\alpha }$ (1)	0.5725	0.543875	0.601125	0.51525	0.62975
Parameter $\bar{\vartheta }$ (1)	2.165	2.05675	2.27325	1.9485	2.3815
Porosity, ${\bar{\varepsilon }}_{\mathrm{ITZ}}$ (1)	0.55	0.5225	0.5775	0.495	0.605
Width of the transition layer, ${\bar{w}}_{t}$ (μm)	75	71.25	78.75	67.5	82.5
Critical time, ${\bar{t}}_{\mathrm{cr}}$ (h)	43	40.85	45.15	38.7	47.3

**Table 4:** Elastic and strength parameters of concrete.

Material Parameter	Value
Modulus of elasticity, E (GPa)	35.605
Poisson’s ratio, ν (−)	0.2
$\mathrm{Uniaxial}\;\mathrm{tensile}\;\mathrm{strength},\;f_{t}\;\left(\mathrm{MPa}\right)$	3.36
$\mathrm{Uniaxial}\;\mathrm{compressive}\;\mathrm{strength},\;f_{c}\;\left(\mathrm{MPa}\right)$	45.4
$\mathrm{Biaxial}\;\mathrm{compressive}\;\mathrm{strength},\;f_{\mathrm{bc}}=1.15{\;f}_{c}\;\left(\mathrm{MPa}\right)$	1.15·45.4

**Table 5:** Elastic and strength parameters of steel.

Material Parameter	Value
$\mathrm{Young}’s\;\mathrm{modulus},\;E_{s}\;\left(\mathrm{GPa}\right)$	200
$\mathrm{Poisson}’s\;\mathrm{ratio},\;\nu_{s}\;\left(1\right)$	0.3
$\mathrm{Yield}\;\mathrm{strength},\;f_{y}$ (MPa)	235

**Table 6:** Inelastic parameters of the MWR material model (ATENA, CC3DnonLin Cementitious2).

**Material Parameter**	**Value**
Onset of crushing, $f_{c0}$ (MPa)	−7.05
Plastic strain, $\varepsilon_{\mathrm{cp}}$	−0.00123
Critical compressive displacement, $w_{d}$ (m)	−0.0005
Compressive strength reduction, c, (1)	0.8
Fracture energy, $G_{F}\;\left(N/m\right)$	145

**Table 7:** Inelastic parameters of the MW material model with HSD2 (ANSYS).

Material Parameter	Value	Material Parameter	Value
Fracture energy, $G_{\mathrm{ft}}\;\left(N/m\right)$	145	$\Omega_{\mathrm{ci}}\;{\left(1\right)}^{*}$	0.16
Dilation angle, ψ (Deg)	20	$\Omega_{\mathrm{cu}}\;{\left(1\right)}^{*}$	0.85
$\kappa_{\mathrm{cm}}\;{\left(1\right)}^{*}$	0.00123	$\Omega_{\mathrm{cr}}\;{\left(1\right)}^{*}$	0.2
$\kappa_{\mathrm{cu}}\;{\left(1\right)}^{*}$	0.003	$\Omega_{\mathrm{tr}}\;{\left(1\right)}^{*}$	0.1

* Parameter characterizing the hardening/softening curve of the HSD2 model under compression and tension.

**Table 8:** Inelastic parameters of the EDM material model (ANSYS).

Material Parameter	Value
$\mathrm{Damage}\;\mathrm{threshold},\;\gamma_{0}\cdot 10^{6}\;\left(1\right)$	108.52
Acceptable degradation of the material, α (1)	0.96
Damage evolution constant, β (1)	150
$\mathrm{Gradient}\;\mathrm{parameter},\;c\;\left({\mathrm{mm}}^{2}\right)$	5

**Table 9:** Parameters of a steel–concrete contact bond model in ANSYS and ATENA programs.

Contact Parameter	Value
Coefficient of friction, μ (1)	1.0
$\mathrm{Normal}\;\mathrm{contact}\;\mathrm{stiffness},\;K_{n}\cdot 10^{-8}\;\left(\mathrm{MN}/m^{3}\right)$	4.11
$\mathrm{Tan}\mathrm{gent}\;\mathrm{contact}\;\mathrm{stiffness},\;K_{t}\cdot 10^{-8}\;\left(\mathrm{MN}/m^{3}\right)\;$	4.11
$\mathrm{Maximum}\;\mathrm{allowable}\;\mathrm{shear}\;\mathrm{stress},\;\tau_{\max}$ (MPa)	$1\cdot 10^{20}$

**Table 10:** Percentage deviation of the results of computer calculations from the average values of the results obtained on the basis of experimental research.

Model	Elongation (mm)	Displacement Difference (mm)	Percentage Deviation from the Mean (%)
Model MWR	0.74	0.03	4.22
Model MW	0.65	0.06	8.4
Model EDM	0.56	0.15	21.1
Experimental study (avg)	0.71	-	-

**Table 11:** Percentage deviation (derived for the affine approach).

Model	Uncertainty $\mu_{\%}$ (%)	Min Time $t_{min}$ (h)	Max Time $t_{max}$ (h)	Time Increment ΔT (h)	Approximate Deviation from $t_{last}$ (%)
Model MWR	0	160.56	160.56	0	0
Model MW	0	178.08	178.08	0	0
Model EDM	0	203.61	203.61	0	0
Model MWR	10	131.75	209.07	77.31	25.2
Model MW	10	141.73	238.15	96.41	31.4
Model EDM	10	159.95	282.63	122.69	40.0
Model MWR	20	109.21	295.84	186.64	60.8
Model MW	20	114.14	307.00	215.86	62.8
Model EDM	20	130.19	307.00	199.81	70.3
Experiment	-	116	307	191.00	65.1

## Data Availability

The data presented in this study are only available from the authors upon reasonable request.

## References

[1] Austin S.A., Lyons R., Ing M. (2004). Electrochemical behaviour of steel reinforced concrete during accelerated corrosion testing. Corrosion.

[2] Sola E., Ožbolt J., Balabanić G., Mira Z.M. (2019). Experimental and numerical study of accelerated corrosion of steel reinforcement in concrete: Transport of corrosion products. Cem. Concr. Res..

[3] Bhalgamiya S., Tivadi G., Jethva M. (2018). Techniques for accelerated corrosion test of steel concrete for determine durability. Int. Res. J. Eng. Technol..

[4] Zhang W., Chen J., Luo X. (2019). Effects of impressed current density on corrosion induced cracking of concrete cover. Constr. Build. Mater..

[5] Bentur A., Alexander M.G. (2000). A review of the work of the RILEM TC 159-ETC: Engineering of the interfacial transition zone in cementitious composites. Mater. Struct..

[6] Michel A., Pease B.J., Peterová A., Geiker M.R., Stang H., Thybo A.E.A. (2014). Penetration of corrosion products and corrosion-induced cracking in reinforced cementitious materials: Experimental investigations and numerical simulations. Cem. Concr. Compos..

[7] Jamali A., Angst U., Adey B., Elsener B. (2013). Modeling of corrosion-induced concrete cover cracking: A critical analysis. Constr. Build. Mater..

[8] Ožbolt J., Oršanic F., Gojko B., Kušte M. (2012). Modeling damage in concrete caused by corrosion of reinforcement: Coupled 3D FE model. Int. J. Fract..

[9] Cao C., Cheung M.M.S. (2014). Non-uniform rust expansion for chloride-induced pitting corrosion in RC structures. Constr. Build. Mater..

[10] German M., Pamin J. (2015). FEM simulations of cracking in RC beams due to corrosion progress. Arch. Civ. Mech. Eng..

[11] Krykowski T., Jaśniok T., Recha F., Karolak M. (2020). A Cracking Model for Reinforced Concrete Cover, Taking Account of the Accumulation of Corrosion Products in the ITZ Layer, and Including Computational and Experimental Verification. Materials.

[12] Yurkova K., Krykowski T. (2023). Modeling of Cracking of the Concrete Cover Taking in to Account the Coupled Diffusion/Mechanical Process. Comput. Assist. Methods Eng. Sci..

[13] Allan M.L. (1995). Probability of corrosion induced cracking in reinforced concrete. Cem. Concr. Res..

[14] Bhargava K., Mori Y., Ghosh A.K. (2011). Time-dependent reliability of corrosion-affected RC beams—Part 1: Estimation of time-dependent strengths and associated variability. Nucl. Eng. Des..

[15] Bhargava K., Mori Y., Ghosh A.K. (2011). Time-dependent reliability of corrosion-affected RC beams. Part 2: Estimation of time-dependent failure probability. Nucl. Eng. Des..

[16] Bhargava K., Mori Y., Ghosh A.K. (2011). Time-dependent reliability of corrosion-affected RC beams. Part 3: Effect of corrosion initiation time and its variability on time-dependent failure probability. Nucl. Eng. Des..

[17] Papakonstantinou K.G., Shinozuka M. (2013). Probabilistic model for steel corrosion in reinforced concrete structures of large dimensions considering crack effects. Eng. Struct..

[18] Bastidas-Arteaga E., Sánchez-Silva M., Chateauneuf A., Silva M.R. (2008). Coupled reliability model of biodeterioration, chloride ingress and cracking for reinforced concrete structures. Struct. Saf..

[19] Bastidas-Arteaga E. (2018). Reliability of reinforced concrete structures subjected to corrosion-fatigue and climate change. Int. J. Concr. Struct. Mater..

[20] Hamdia K.M., Msekh M.A., Silani M., Thai T.Q., Budarapu P.R., Rabczuk T. (2019). Assessment of computational fracture models using Bayesian method. Eng. Fract. Mech..

[21] Górski J. (2006). Non-Linear Models of Structures with Random Geometric and Material Imperfections Simulation-Based Approach.

[22] Dijkman J., van Haeringen H., de Lange S. (1983). Fuzzy numbers. J. Math. Anal. Appl..

[23] Richtmyer R.D., Moore R.E. (1968). Interval analysis. Math. Comput..

[24] Stolfi J., de Figueiredo L.H. Self-validated numerical methods and applications. Proceedings of the Monograph for 21st Brazilian Mathematics Colloquium, IMPA.

[25] Qiu Z., Yang D., Elishakoff I. (2008). Probabilistic interval reliability of structural systems. Int. J. Solids Struct..

[26] Degrauwe D., Lombaert G., De Roeck G. (2010). Improving interval analysis in finite element calculations by means of affine arithmetic. Comput. Struct..

[27] Sofi A., Romeo E. (2016). A novel Interval Finite Element Method based on the improved interval analysis. Comput. Methods Appl. Mech. Eng..

[28] Krykowski T. (2020). The application of Affine/Interval Algebra to determine the time of concrete cover damage in reinforced concrete due to corrosion. Comput. Assist. Methods Eng. Sci..

[29] Recha F. (2021). Modeling of the Degradation of Reinforced Concrete Elements as a Result of Reinforcement Corrosion. Ph.D. Thesis.

[30] Rump S.M., Kashiwagi M. (2015). Implementation and improvements of affine arithmetic. Nonlinear Theory Its Appl. IEICE.

[31] Metropolis N., Ulam S. (1949). The Monte Carlo method. J. Am. Stat. Assoc..

[32] Bilotta G. (2008). Self-verified extension of affine arithmetic to arbitrary order. Le Mat..

[33] De Figueiredo L.H., Stolfi J. (2004). Affine arithmetic: Concepts and applications. Numer. Algorithms.

[34] El-Owny H. (2006). Hansen’s Generalized Interval Arithmetic Realizedin C-XSC. Bergische Universität Wuppertal. http://www2.math.uni-wuppertal.de/org/WRST/preprints/prep_06_2.pdf.

[35] Manson G. (2005). Calculating frequency response functions for uncertain systems using complex affine analysis. J. Sound Vib..

[36] Pantazopoulou S.J., Papoulia K.D. (2001). Modeling cover-cracking due to reinforcement corrosion in rc structures. J. Eng. Mech..

[37] Wieczorek B., Krykowski T. (2017). Application of damage mechanics rules to evaluate the growth of corrosive deformations in transition layer. Corros. Prot..

[38] Ansys Inc. (2023). Chapter 4: Nonlinear Material Properties. Ansys Mechanical 2023 Help System.

[39] Menetrey P., Willam K.J. (1995). Triaxial failure criterion for concrete and its generalization. ACI Struct. J..

[40] Menetrey P. (1994). Numerical Analysis of Punching Failure in Reinforced Concrete Structures. Ph.D. Thesis.

[41] Červenka V., Jendele L., Červenka J. (2016). Atena Program Documentation, Part 1, Theory.

[42] Červenka J., Papanikolaou V.K. (2008). Three dimensional combined fracture-plastic material model for concrete. Int. J. Plast..

[43] Zreid I., Kaliske M. (2014). Regularization of microplane damage models using an implicit gradient enhancement. Int. J. Solids Struct..

